# Relationships between body size attitudes and body image of 4-year-old boys and girls, and attitudes of their fathers and mothers

**DOI:** 10.1186/s40337-015-0048-0

**Published:** 2015-04-10

**Authors:** Stephanie R Damiano, Karen J Gregg, Emma C Spiel, Siân A McLean, Eleanor H Wertheim, Susan J Paxton

**Affiliations:** School of Psychology and Public Health, La Trobe University, Melbourne, Vic 3086 Australia

**Keywords:** Boys, Girls, Body image, Body size attitudes, Mother, Father

## Abstract

**Background:**

Body size attitudes and body image form early in life, and understanding the factors that may be related to the development of such attitudes is important to design effective body dissatisfaction and disordered eating prevention interventions. This study explored how fathers’ and mothers’ body size attitudes, body dissatisfaction, and dietary restraint are associated with the body size attitudes and body image of their 4-year-old sons and daughters.

**Methods:**

Participants were 279 4-year-old children (46% boys) and their parents. Children were interviewed and parents completed questionnaires assessing their body size attitudes and related behaviours.

**Results:**

Socially prescribed stereotypical body size attitudes were evident in 4-year-old boys and girls; however, prevalence of body dissatisfaction was low in this sample. Correlation analyses revealed that boys’ body size attitudes were associated with a number of paternal body image variables. In boys, attributing negative characteristics to larger figures and positive characteristics to thinner figures were associated with fathers having more negative attitudes towards obese persons. Attributing positive characteristics to larger figures by boys was associated with greater levels of paternal dietary restraint. In girls, attributing positive characteristics to thinner figures was only associated with greater maternal dietary restraint.

**Conclusions:**

Findings suggest the possibility that fathers’ body size attitudes may be particularly important in establishing body size attitudes in their sons. Further research is necessary to better understand the role of fathers in the development of children’s body size attitudes.

## Background

A growing literature suggests that children’s body size attitudes and body image are formed in early childhood. Socially prescribed stereotypical body size attitudes, that is, attributing negative characteristics to larger body sizes and positive characteristics to thinner body sizes, have been consistently observed among very young children [1-4]. Such stereotypical body size attitudes have been associated with increased weight stigma and engaging in teasing and discrimination, and being a recipient of such behaviours may increase children’s vulnerability to experiencing low self-esteem, depression, and body dissatisfaction [5,6]. A preference for thin bodies, body dissatisfaction, and dieting awareness have also been observed in pre-schoolers [7] and young children aged five to eight years [8]; which are potential risk factors for the development of disordered eating [9]. Thus, it is essential to identify environmental factors related to the early development of body size attitudes and body image so that effective prevention interventions can be developed to prevent the later onset of body dissatisfaction and eating problems.

In addition to biological and psychological factors, modifiable social factors that include parent influences have been a major focus of theoretical and empirical investigations into the development of body image [10]. In young children, it has been proposed that parents and family play a formative role in shaping the development of body size attitudes and body image, as the family may act as a filter that regulates the child’s exposure to media and prevailing cultural ideals [11]. Parents have been proposed to influence their children in numerous ways: their expressed evaluations about their own and each other’s bodies may serve as models for children to critique themselves and others [12,13]; their engagement in behaviours, such as exercising and dieting activities, may model the importance of adhering to cultural body size ideals [8,14]; and their direct instruction, comments, appearance criticism, and teasing may reinforce cultural body ideals and body size stigma [8,15].

In pre-adolescent and early adolescent children, research is broadly supportive of relationships between mothers’ body size attitudes and their children’s, especially daughters’, attitudes. For example, Davison and Birch [11] found that 9-year-old girls were more likely to express negative attitudes about obesity and obese persons when they perceived their mother to be concerned about her own and her child’s weight. Research has also found positive associations between mothers’ self-reported negative obesity stereotypes and the self-reported stereotypes of their pre-adolescent boys and girls [16,17]. Similarly, associations have been observed between mothers’ self-reported body image and related behaviours, such as weight-related comments, dieting, and encouragement of child to diet, and pre- and early adolescent boys’ and girls’ body dissatisfaction, desire to be thinner, and body ideals [18,19].

Only a small number of studies have examined relationships between mothers’ body size attitudes and body image and the body image of their preschool and early school-age children, despite previous research suggesting that parent influences would be especially pertinent at this early age [3,12]. Specifically, Holub, Tan, and Patel [12] found that mothers’ dislike of overweight children and personal fear of fat were positively associated with young children’s negative attitudes about an overweight figure. Moreover, Spiel, Paxton, and Yager [3] observed that mothers’ internalization of the thin ideal was associated with young children selecting thinner figures for positive characteristics. A further study of 5- to 8-year-olds showed that a child’s perception of their mother’s body dissatisfaction was correlated with the child’s body dissatisfaction, as measured by the discrepancy between current and ideal figure selections, in both girls and boys [8]. Although these studies were cross-sectional in nature they suggest that mothers may influence their children’s body size attitudes from a young age.

Relationships between fathers’ and their children’s, especially sons’, body size attitudes and body image have seldom been investigated. Theoretical and empirical literature suggests that fathers influence their children in numerous ways. In particular, children may mirror their same-sex parent’s behaviours, and research findings that boys learn gender roles from their fathers support this contention (e.g., [20,21]). Consequently, it might be expected that young sons would reflect the body size attitudes and body image of their fathers. Research investigating these relationships has mainly been conducted with pre- and early adolescent children and findings have been mixed. McCabe and Ricciardelli [22] reported that, in adolescent boys, direct influences, such as pressure from fathers to lose weight and increase muscle, prospectively predicted use of strategies to lose weight, whilst pressure from both mother and father to increase muscle predicted use of strategies to increase muscle. In addition, negative comments about weight by fathers have been shown to predict binge eating in their sons [23]. On the other hand, indirect influences, such as fathers’ body dissatisfaction, has been shown to predict thin body preoccupation in 11-year-old girls but not boys [24]. Further, fathers’ dieting has not been shown to be associated with early adolescent sons’ or daughters’ body related attitudes [19]. The inconsistency in these findings suggests that more research is necessary to investigate how fathers’ attitudes and behaviours may relate to those of their children, and in particular to understand these potential relationships in the formative years of children’s body size attitudes and body image.

Despite this need, we are unaware of any research that has examined relationships between fathers’ body size attitudes, body dissatisfaction, or dieting and the body size attitudes and body image of their preschool children, despite this being an age at which the influence of parents is particularly strong. Given the dearth of existing knowledge in this area, better understanding the potential influences on the development of body size attitudes and body image in preschool age children is important as these are the formative years for these attitudes [2,3]. The transmission of attitudes of fathers to their sons and of mothers to their daughters may be of particular importance in considering a gender-linked model.

In this study, we aimed to address these research gaps. The first aim of this study was to replicate previous observations of positive and negative body size attitudes in young children, and to better understand the presence of body dissatisfaction in 4-year-old boys and girls. The second aim was to extend our understanding of body size attitudes in young children by exploring parental factors related to the body size attitudes and body image of 4-year-old boys and girls. Specifically, we explored corresponding constructs in parents and children, including body size attitudes and body dissatisfaction. Despite not assessing dieting in the children, due to measurement difficulties at this young age, we explored parental dietary restraint due to the important relationships with child outcomes in previous research (e.g., [18]). We hypothesised that 4-year-old boys and girls would attribute negative characteristics to larger figures and positive characteristics to thinner figures. Further, we hypothesised that children’s attribution of negative characteristics to larger figures and positive characteristics to thinner figures would be associated with greater parental anti-fat attitudes, body dissatisfaction, and dietary restraint. In addition, we aimed to examine the presence of child body dissatisfaction (indicated by the discrepancy between their perceived current and ideal figure) and the relationships between child body dissatisfaction and parental body dissatisfaction, anti-fat attitudes, and dietary restraint. Given the limited previous research investigating very young children’s body dissatisfaction and parental correlates, we considered that this part of the investigation was exploratory, thus, no specific hypothesis was posed. Finally, we anticipated that hypothesized relationships would be stronger between same-sex parent–child dyads than between opposite-sex dyads.

## Methods

### Participants

Participants were 279 4-year-old children (boys, *n* = 127; girls, *n* = 152; *M*_*age*_ = 4.49 years, *SD* = 0.35), 205 of their fathers (*M*_*age*_ = 40.22 years, *SD* = 5.35), and 270 mothers (*M*_*age*_ = 38.61 years, *SD* = 4.64) recruited in Melbourne, Australia, using strategies including advertisements placed in childcare centres and playgroups, and advertising at a stall at a local animal farm. Participants joined a longitudinal study, named the Children’s Body Image Development Study, when children were three-years old (*N* = 295). To be involved in the study, it was necessary that children were three-year-olds at baseline, parents were over the age of 18 years, and that families lived in Melbourne, Australia. This study examines data from the second assessment (16 children from the baseline sample moved and did not participate in the second assessment). Postcode data indicated 58% of families lived in high, 32% in average, and 10% in disadvantaged socioeconomic areas [25]. Parents were generally well-educated with 77.1% of mothers and 65.4% of fathers reporting completion of a university degree.

### Measures

#### Demographics

Parents completed a questionnaire that included age, education, and postcode, and weight and height from which Body Mass Index (BMI) was derived. Anthropometric measures were also collected for children; the interviewer weighed and measured the children. BMI was calculated and converted into a standardized BMI *z*-score adjusting for age and gender using the World Health Organization’s (WHO) child growth standards software [26].

### Child body size attitudes and ideals

#### Body size attitudes

Children’s body size attitudes, reflecting weight stereotypes about children of different body sizes, were assessed with the Children's Body Size Attitudes Scale, an adaptation of the play based measure described by Spiel et al. [3] for use in preschool children. This instrument was derived from two previously used measures of weight bias in young children: one that asked children to select a body figure size to illustrate a child with positive or negative qualities described in a short story format [27], and one that asked children to select a body figure size to illustrate children in particular social situations using a structured interview format [28]. The array of body figures used was adapted from the child figure rating scale of Tiggemann and Pennington [29]. Five silhouette figures (one set for girls, one set for boys), 20 cm in height and ranging in size (1 (*very thin*), 2 (*thinner than average*), 3 (*average*), 4 (*fatter than average*), and 5 (*very fat*)), were created using coloured felt fabric, and children were told that the body figures were their age. Children were also able to choose the hair colour that closely matched their own, to assist children in identifying with the figures.

Following pilot testing to identify the most appropriate questions for this age group, to assess negative body size attitudes, children were asked to respond to eight questions. First they were asked to select a body figure in response to the following questions, similar to Birbeck and Drummond [28]: which figure they would not invite to their birthday party; which has no friends to play with; which plays all by him/herself; which other children do not really like; and which gets teased by other children? Children were also read three age-appropriate illustrated stories about one child who was *mean*, one who was *naughty*, and one who was *rude.* Children were then asked to select the figure to represent the child in each story [3]. For example, “One of these little boys/girls is very mean. This little boy/girl breaks the other children’s crayons, squashes their lunches, and splatters paint over their clothes. He/she even pushes other children if they are in his/her way. Can you guess which one of these [interviewer pointing to figure rating scale] is the mean boy/girl?” The mean score for selected body figures was calculated; higher scores indicated children selected fatter figures for negative characteristics.

Following pilot testing to identify the most appropriate questions for this age group, to assess positive body size attitudes, children were asked to select a body figure that corresponded to two questions: which figure they would invite to their birthday party; and which has lots of friends to play with. Children were also read four brief age-appropriate stories about children who were *good*, *happy*, *fun*, and *clever*, and selected a figure to correspond to each [3]. For example, “One of these little boys/girls is a good boy/girl. This little boy/girl brushes his/her teeth every morning and every night and always keeps his/her room clean and tidy. He/she would never say something to his/her mummy or daddy that was not true. Can you guess which one of these is the good boy/girl?” The mean score for selected body figures was calculated; lower scores indicated children selected thinner figures for positive characteristics.

Cronbach’s alphas for the 8-item negative and 6-item positive body size attitudes scales were .63 and .60 respectively, indicating moderate levels of reliability for the scale scores [30]. Similar measures of stereotypical body size attitudes in children aged three to five [3] and children aged three to six [1] have shown comparable levels of internal consistency ranging between .54 - .67. Supporting the construct validity of these measures in this sample, the positive and negative body size attitudes scales showed a strong negative correlation (*r* = −.57, *p* < .001). Further supporting construct validity, the correlation between the positive body size attitudes scale scores and responses to a single question asking whether children wanted to look like body Figure 1 (*very thin*) was examined and found to be significant, *r*_*b*_ = .23, *p* = .03. Similarly, attributing negative characteristics to fatter figures was correlated with not wanting to look like body figure 5 (*very fat*), *r*_*b*_ = .26, *p* = .04.

A pilot study was conducted with 62 children with a mean age of 4.65 years (*SD* = 0.89) to determine test-retest reliability of these measures of body size attitudes; with assessments two weeks apart [31]. Intraclass Correlations (ICCs) showed good agreement between test (T1) and retest (T2) scores for positive body size attitudes (*M*_*T1*_ = 2.93, *SD*_*T1*_ = 0.71; *M*_*T2*_ = 2.95, *SD*_*T2*_ = 0.71; *ICC* = .70, *p* < .001) and negative body size attitudes (*M*_*T1*_ = 3.88, *SD*_*T1*_ = 0.74; *M*_*T2*_ = 3.90, *SD*_*T2*_ = 0.74; *ICC* = .70, *p* < .001) [31], according to criteria outlined by McDowell [32]. Paired samples *t*-tests revealed no significant differences in children’s reports of positive (*t* (61) = −0.20, *p* = .84) or negative (*t* (61) = −0.32, *p* = .75) body size attitudes across the two testing points, indicating that children’s reports of body size attitudes were temporally reliable [31].

#### Body dissatisfaction

To assess children’s body dissatisfaction we evaluated the discrepancy between their perceived current and ideal size. Using the same five figure array described above, children selected the body figure they perceived to be most like themselves (current) and the one they would most like to be (ideal). A current-ideal discrepancy score was calculated as current figure minus ideal figure, a well-documented method for assessing body dissatisfaction in older children (e.g, [8,33]) and children aged between four and six years [34]. Scores ranged from −4 to 4. A score of 0 indicated body satisfaction, positive scores indicated a desire to be thinner, and negative scores a desire to be larger.

From the unpublished data in the pilot study described above [31], ICC analyses indicated significant agreement between test (T1) and retest (T2) for current figure selection (*M*_*T1*_ = 2.35, *SD*_*T1*_ = 1.46; *M*_*T2*_ = 2.50, *SD*_*T2*_ = 1.36; *ICC* = .58, *p* = .03), and ideal figure selection (*M*_*T1*_ = 2.98, *SD*_*T1*_ = 1.40; *M*_*T2*_ = 2.53, *SD*_*T2*_ = 1.41; *ICC* = .34, *p* = .05). Further supporting the reliability of children’s responses, Wilcoxon Signed Rank Tests revealed no significant changes across time for the current figure (*z* = −.42, *p* = .68) and ideal figure (*z* = −1.34, *p* = .18) selections [31].

### Parent body size attitudes, body image, and eating behaviours

Parents’ negative attitudes about obese people were measured using five items from the Attitudes Towards Obese People scale [35], two from the Beliefs About Obese People scale [35], and two from Crandall’s [36] Willpower scale. These nine items were selected as they directly measure a stigmatizing attitude about individuals who are obese (e.g., “Fat people tend to be obese pretty much through their own fault”). The items from these scales that were not included measured additional constructs, such as self-esteem, that were not considered relevant for this study. Principal components analysis indicated that these items loaded on one component, which we named Negative Attitudes Towards Obese Persons. Responses are made on a 100mm visual analogue scale ranging from 0 (*strongly disagree*) to 100 (*strongly agree*), as pilot work indicated this format was less confronting than asking parents to indicate specific categories of agreement for their stigmatizing beliefs about obese individuals. The mean item score was calculated (higher scores = more stigmatizing attitudes). Cronbach’s alphas were satisfactory: mothers α = .73; fathers α = .73. (Scale items are available from authors).

Three subscales of the Body Attitudes Questionnaire (BAQ; [37]) were combined to assess parent body dissatisfaction, the 13-item Feeling Fat, the 8-item Disparagement, and the 8-item Salience subscales (e.g., “If I catch sight of myself in the mirror or shop window it makes me feel bad about my shape”). Items are scored from 1 (*strongly disagree*) to 5 (*strongly agree*). Item scores were combined to provide a single comprehensive score for body dissatisfaction (higher scores = greater dissatisfaction). This was deemed suitable given that the Cronbach’s alphas in our sample were satisfactory: mothers α = .93; fathers α = .92.

The 10-item Restraint subscale of the Dutch Eating Behaviors Questionnaire [38] was used to assess frequency of parental dietary restraint (e.g., “Do you deliberately eat less in order not to become heavier?”). This measure assesses cognitive restraint rather than dieting behaviours per se [39]. Items are rated from 1 (*never*) to 5 (*very often*), and the mean item score was calculated (higher scores = more restrained eating). Cronbach’s alphas in our sample were satisfactory: α = .90 for both mothers and fathers.

### Procedure

The La Trobe University Human Ethics Committee provided ethics approval. Parents gave written consent for their own and their child’s involvement, and the child gave verbal assent prior to the interview. Interviews were conducted at the child’s home with a parent present. Where possible, both the child’s mother and father completed self-report measures prior to, during, or soon after the interview. At completion, children were given a sticker and families an AU$10 shopping voucher and the opportunity to win further shopping vouchers in a prize draw.

### Statistical analyses

Children’s current and ideal figure selection were not normally distributed, thus, non-parametric analyses were conducted for these scales. All other scales were normally distributed, thus, parametric analyses were conducted. Paired samples *t*-tests (for normally distributed variables) and Wilcoxon Signed Rank Tests (for non-normally distributed variables) were conducted to explore the children’s figure selections for negative and positive characteristics, and body image. Pearson (for normally distributed variables) and Spearman (for non-normally distributed variables) correlation analyses (two–tailed) were conducted to determine associations between body size attitudes, body image, and eating behaviours of fathers and mothers and the body size attitudes and body image of their sons and daughters, separately. To identify the variance in child body size attitudes and current-ideal discrepancy accounted for by the group of parent variables that were correlated significantly (*p* < .05) with child variables, these variables were entered into multiple regression analyses for boys and girls separately.

## Results

### Sample characteristics

Among the 274 (98.2%) children for whom complete height and weight information was available, BMI ranged between 11.11 and 24.46 (*M* = 15.56, *SD* = 1.56) and BMI*z* for age and gender ranged between −3.60 and 5.29 (*M* = 0.15, *SD* = 1.08). According to WHO [26] BMI-for-age criteria, 78.8% of children were considered healthy weight, 3.3% underweight, 9.9% overweight, and 8.0% obese. For the 267 (98.9%) mothers who provided anthropometric information, BMI ranged from 17.41 to 44.63 (*M* = 24.72, *SD* = 4.81), with 4.1% underweight, 56.6% normal weight, 27.0% overweight, and 12.4% obese according to WHO criteria [40]. For the 199 (97.1%) fathers who provided anthropometric information, BMI ranged from 17.87 to 39.05 (*M* = 26.41, *SD* = 3.72), with 0.5% underweight, 37.7% normal weight, 46.2% overweight, and 15.6% obese according to WHO criteria [40].

### Child body size attitudes and body image

Table 1 shows descriptive information for body size attitudes and body image for all participants, in addition to dietary restraint of parents. The mean body figure size selected for negative characteristics by both boys and girls was significantly larger than that selected for positive characteristics; boys: *t*(126) = 7.09, *p* < .001; girls: *t*(151) = 9.79, *p* < .001. As seen in Table 2, boys and girls most frequently reported body figures 1, 2, and 3 as their perceived current and ideal sizes. Wilcoxon Signed Rank Tests revealed that the mean current figure selection was significantly smaller than the ideal figure selection (boys: *z* = −3.23, *p* = .001; girls: *z* = −2.23, *p* = .026), although as seen in Table 1, the ideal figure was still on average smaller than the average size (body figure 3 in the figure rating scale). In other words, children wanted to be larger than they perceived themselves to be, but they did not want to be the fattest figure. Additional Wilcoxon Signed Rank Tests revealed that the mean figure selection for current figure was also significantly thinner than the figure selected for positive characteristics for both boys and girls (boys: *z* = −5.23, *p* < .001; girls: *z* = −4.23, *p* < .001).

**Table 1:** **Mean scores of child and parent body size attitudes and body image**

**Variable**	***M***	***SD***	**Range**	***M***	***SD***	**Range**
	**Boys**			**Girls**		
Figure for negative characteristics	3.49	0.76	1.63 – 5.00	3.57	0.76	1.57 – 5.00
Figure for positive characteristics	2.65	0.75	1.00 – 4.67	2.54	0.72	1.00 – 4.67
Current figure	2.09	1.22	1.00 – 5.00	2.13	1.11	1.00 – 5.00
Ideal figure	2.36	1.32	1.00 – 5.00	2.31	1.16	1.00 – 5.00
Current – Ideal discrepancy	−0.27	0.96	−4.00 – 4.00	−0.19	1.00	−4.00 – 3.00
	**Fathers**			**Mothers**		
Negative attitudes towards obese persons	57.87	12.70	25.56 – 91.37	53.85	11.83	21.64 – 85.93
Body dissatisfaction	20.01	5.35	10.33 – 37.67	23.64	5.76	11.67 – 42.00
Dietary restraint	2.08	0.73	1.00 – 4.00	2.54	0.75	1.00 – 5.00

*Note:* Boys *n* = 127; Girls *n* = 152; Fathers *N* = 205; Mothers *N* = 270.

**Table 2:** **Percentage of boys and girls selecting figure sizes for perceived current and ideal body size**

**Variable**	**Gender**	**Body figure 1**	**Body figure 2**	**Body figure 3**	**Body figure 4**	**Body figure 5**
		**% (** ***n*** **)**	**% (** ***n*** **)**	**% (** ***n*** **)**	**% (** ***n*** **)**	**% (** ***n*** **)**
Current figure	Boys	42.9 (54)	24.6 (31)	21.4 (27)	3.2 (4)	7.9 (10)
	(*n* = 126)					
	Girls	36.2 (55)	30.3 (46)	23.0 (35)	5.9 (9)	4.6 (7)
	(*n* = 152)					
Ideal figure	Boys	34.9 (44)	23.0 (29)	24.6 (31)	6.4 (8)	11.1 (14)
	(*n* = 126)					
	Girls	29.1 (44)	31.8 (48)	23.8 (36)	9.3 (14)	6.0 (9)
	(*n* = 151)					

*Note:* Body Figure 1 = thinnest figure; Body Figure 5 = fattest figure.

Of boys, 27.8% (*n* = 35) desired to be a different size from their perceived current figure, of which 22.9% (*n* = 8) had a thinner ideal than current figure, and 77.1% (*n* = 27) had a larger ideal than current figure. Of girls, 37.7% (*n* = 57) desired to be a different size, of which 35.1% (*n* = 20) had a thinner ideal than current figure, and 64.9% (*n* = 37) had a larger ideal than current figure.

For both boys and girls, a larger perceived current figure was associated with a larger ideal figure (boys *r* = .74, *p* < .001; girls *r* = .59, *p* < .001), larger figure for positive characteristics (boys *r* = .33, *p* < .001, girls *r* = .36, *p* < .001), and thinner figure for negative characteristics (boys *r* = −.22, *p* = .02; girls *r* = −.38, *p* < .001). In both boys and girls, BMI*z* was not related to any of the assessed body size attitudes; consequently, child BMI*z* was not controlled for in analyses.

### Relationships between boys’ and parents’ body size attitudes and body image variables

#### Boys’ figure selected for negative characteristics

Table 3 shows correlations between boys’ body image variables and parent body image and eating variables. Consistent with our hypothesis, a larger figure selected for negative characteristics was correlated with greater paternal anti-fat attitudes. This was the only significant correlation and regression analyses indicated that paternal anti-fat attitudes accounted for 9% of variance in boys’ figure selection for negative characteristics, *F*(1, 87) = 8.65, *p* = .004 (see Table 4).

**Table 3:** **Correlations between boys’ and parents’ body size attitudes and body image variables**

**Predictor**		**Figure for negative characteristics**	**Figure for positive characteristics**	**Current – Ideal discrepancy**
Parent BMI	Mother	.01	.01	.01
	Father	-.02	.17	.03
Negative attitudes towards obese	Mother	.15	-.14	-.06
	Father	.30**	-.34**	.06
Body dissatisfaction	Mother	.06	-.04	.16
	Father	-.11	.16	-.08
Dietary restraint	Mother	.09	-.03	.05
	Father	-.15	.22*	-.21

*p < .05 **p < .01.

**Table 4:** **Summary of regression analyses of parental predictors of boys’ body size attitudes and body image variables**

**Predictor**	***R*** ^***2***^	***r***	***B***	***SE***_***B***_	***β***	
*DV: Figure for negative characteristics*						
Paternal negative attitudes towards obese persons		.30**	.02	.01	.30**	
	.09					
*DV: Figure for positive characteristics*						
Paternal dietary restraint		.22*	.25	.12	.20*	
Paternal negative attitudes towards obese persons		-.34**	-.02	.01	-.33**	
	.16					

*p < .05 **p < .01.

#### Boys’ figure selected for positive characteristics

In boys, a thinner figure selected for positive characteristics was, as expected, associated with greater paternal anti-fat attitudes. Contrary to expectations, a larger figure selected for positive characteristics was associated with greater levels of paternal dietary restraint. When these two variables were entered into a multiple regression the model explained 16% of variance in figure size selected for positive characteristics, *F*(2, 86) = 8.04, *p* = .001. Paternal dietary restraint and negative attitudes towards obese persons were both significant predictors (Table 4). Given that the relationship between the figure selected by boys for positive characteristics and paternal dietary restraint was not in the anticipated direction, we explored the possibility that paternal BMI moderated this relationship, positing that if fathers were larger, boys may have more positive attitudes towards larger figures. A post hoc hierarchical regression analysis was conducted using centred variables. However, the paternal BMI x dietary restraint interaction was not a significant predictor of figure for positive characteristics, indicating that regardless of paternal BMI there was a positive relationship between figure selected for positive characteristics and paternal dietary restraint.

#### Boys’ body dissatisfaction

No parental variables were correlated with boys’ current-ideal discrepancy score, which may be attributed to the majority of boys (72.2%) being satisfied with their body size.

### Relationships between girls’ and parents’ body size attitudes and body image variables

The only significant relationship observed between girls’ body size attitudes and body image and parental attitudes and behaviours was between girls selecting a thinner figure size for positive characteristics and greater maternal dietary restraint (*r* = −.18, *p* = .03). Regression analyses showed that maternal dietary restraint accounted for 3% of girls’ figure selection for positive characteristics, *F*(1,146) = 4.73, *p* = .031. (Further information is available from authors).

## Discussion

This study is the first to include fathers in addition to mothers in an investigation of relationships between body size attitudes and body image of 4-year-old boys and girls and the attitudes and behaviours of their parents. Our research supports previous observations of the presence of stereotypical positive and negative body size attitudes in young children. In addition, our study provides evidence of the relationships between parental endorsement of stereotypical body size attitudes and those of their young children, and provides some preliminary support for a gender-linked model in which young boys’ attitudes are more strongly associated with the attitudes and behaviours of their father than mother [19].

Boys and girls in our sample demonstrated weight bias that was consistent with stereotypical societal ideals by selecting thinner figures for positive characteristics and fatter figures for negative characteristics. These findings are consistent with previous research that shows that preferences for thin bodies and biases against fatter bodies appear to be present by four years of age [1,3,4,7]. In the present study, when children perceive themselves to be larger, however, they tend to report less extreme body size attitudes. These findings are consistent with that of Holub [1] who observed that young children who perceived that they were heavier held fewer anti-fat attitudes relative to children who perceived they were lighter. Additionally, in the present study, children who perceived that they were heavier reported a larger ideal figure, which at this age may be protective, and is consistent with previous findings in older age groups (e.g., [41]).

It is concerning that at four years old, some boys and girls who perceive they have a larger body size desire to be thinner; potentially setting the stage for early onset negative self-perceptions and weight loss behaviours. Notably, however, the majority of 4-year-old girls and boys were satisfied with their body size. Previous research has established that body dissatisfaction is at much higher prevalence in girls and boys by the time of early adolescence [42]. Future research is required to identify critical periods for body dissatisfaction in childhood and adolescence. The finding of low overall body dissatisfaction in our study presents an interesting contrast to the extent to which children demonstrated weight bias. Tracking prospective relationships between changes in negative and positive body size attitudes towards others and changes in children’s attitudes to their own body image would be a valuable direction for future research.

As hypothesized, in boys, selection of fatter figures for negative characteristics and selection of thinner figures for positive characteristics were associated with greater paternal anti-fat attitudes. These findings suggest that even in very young children, body size attitudes correspond with parental endorsement of social stereotypes surrounding body size. Although causal relationships between variables cannot be assumed from cross-sectional data, a likely explanation is that boys learn negative attitudes about obese persons and positive attitudes about thinner bodies from their parents, especially fathers.

Contrary to our expectations, boys’ selection of larger figures for positive characteristics was associated with more paternal dietary restraint, although the relationship was not strong. We considered that this may have been explained by the influence of a father’s body size on the relationship between paternal dietary restraint and figure selection; however, our findings indicated that paternal BMI did not moderate this relationship at a cross-sectional level. A possible explanation may be that fathers are restraining their eating as a means of reducing body fat, whilst simultaneously desiring an increase in muscle mass, in line with the muscular societal ideal for males (e.g., [43]); thus, boys may be internalising the notion of the lean but larger muscular ideal. It is important that in future research, both fathers’ drive for muscularity and boys’ desire for muscularity are measure to assess this possibility.

Importantly, in girls, the only association between child and parental attitudes was between girls’ selecting thinner figures for positive characteristics and greater maternal dietary restraint. This finding is somewhat consistent with previous research in older girls whereby maternal dietary restraint was associated with girls’ weight concerns [18] and that adolescent girls are more likely to engage in weight loss attempts when their mothers engaged in extreme and presumably more observable weight loss behaviours rather than moderate weight watching and body dissatisfaction [44]. An explanation for the limited findings between mothers and girls in the present study may be that the body size attitudes and body image of girls are being influenced to a greater extent by other sources, such as media, dolls, or peers, from a young age. Consistent with this suggestion is the increased exposure of girls to extremely thin dolls, such as Barbie [45], and cartoons, such as Cinderella, which may convey the societal thin ideal [46].

Interestingly, the findings from this study revealed that for boys, only paternal variables were associated with their body size attitudes, whereas for girls, only maternal variables were associated with their body size attitudes, supporting a gender-linked model of the transmission of body size attitudes. Replication of these findings in future research has a number of important implications for the design and implementation of body image interventions for parents to enhance positive body image in young children. Although fathers are often difficult to engage in interventions for their children, our findings highlight some relationships between fathers’ and their sons’ body size attitudes suggesting the importance of the inclusion of fathers. Mothers are typically seen as primary caregivers, thus, it is thought that they are likely to have more influence on their children; however, the present findings suggest that a father’s influence may also be particularly important at this early stage of development. In addition, parents need to be aware that their body size attitudes and eating behaviours are likely to have an impact on their young children to a greater extent than parents’ own body image. Prevention resources for parents may need to assist them in conveying body size messages that will support positive body image in their children. In light of the relationship between greater maternal dietary restraint and selection of thinner figures to represent positive characteristics, suggesting possible internalization of the societal thin ideal by girls, prevention resources may need to inform mothers that their dietary restraint may be associated with attitudes that make their daughters vulnerable to body image problems in the future. Importantly, replication of the current findings and extension of research to examine the role of other influences, such as sociocultural pressure from sources including media, is necessary to better inform the development of such prevention resources.

These findings must be considered in light of some limitations. This is a cross-sectional study, thus, causal statements about relationships between variables cannot be made. In addition, the variance in children’s body size attitudes accounted for by parental variables was low, suggesting the need to examine further variables that might also be related to young children’s attitudes. Longitudinal studies are required to examine prospective relationships. In 4-year-old children measurement issues also arise. In particular, although calculating the perceived current-ideal discrepancy to indicate level of body dissatisfaction is a well-established method for measuring body dissatisfaction in children as young as 5-years-old [8], it has seldom been used with 4-year-old children. However, Marsh, Ellis, and Craven [47] have shown that 4- and 5-year-old children can reliably report on their appearance-related self-concept, and children’s reports of their current and ideal figure selection have shown to be internally consistent [31]. In future research in young children, it would be valuable to further explore age appropriate measures of body image attitudes. In addition, we do not have a valid and reliable measure of desire to be more muscular but also lean, in >4-year-old boys, a limitation noted by previous researchers (e.g., [22]). Furthermore, in future it would be valuable to include a specific measure of desire for muscularity in fathers, and potentially a measure of perceived muscularity. Finally, our sample was on average better educated and of higher socio-economic status than the general Australian population. Therefore, care needs to be taken in generalizing our results to other samples, either in Australia or around the world.

## Conclusion

This study highlights gender-linked associations between fathers’ negative attitudes towards obese people and the body size attitudes of their 4-year-old sons, as well as the dietary restraint of mothers and the body size attitudes of their daughters. The findings also suggest that paternal attitudes and behaviours are more strongly associated with body size attitudes and body image of boys, than maternal attitudes and behaviours are associated with attitudes of girls at this young age. Nevertheless, maternal dietary restraint was found to be associated with girls’ body size attitudes, specifically the figure selected for positive characteristics, which has important implications for prevention. The findings highlight that body dissatisfaction is not highly prevalent in 4-year-old children, thus, understanding what influences dissatisfaction at this age is difficult to determine. Further research is needed to facilitate understanding of these influences. Our findings suggest that body size attitudes are being formed very early in childhood and are related to the attitudes of their parents. In support of the review by Hart, Cornell, Damiano, and Paxton [48], the current findings suggest there is a need for effective prevention interventions for parents of very young children to encourage parents to create an environment to foster the development of healthy body size attitudes and body image.
